# Emerging Tools for Computer-Aided Diagnosis and Prognostication

**DOI:** 10.4172/2167-0870.1000e117

**Published:** 2014-02-24

**Authors:** Scott Ritter, Kenneth B Margulies

**Affiliations:** Department of Medicine, Division of Cardiovascular Medicine, Cardiovascular Institute, Perelman School of Medicine, University of Pennsylvania

The ability to more accurately predict and prevent disease has the potential to transform clinical practice by improving response to specific treatment regimens and decreasing morbidity and mortality. Part of what limits the accuracy to which we can predict and prevent disease results from our limited understanding of the relationship between clinical presentation and disease progression [1].

Although vast amounts of data are collected at clinical presentation, ranging from macro-scale Magnetic Resonance Imaging (MRI) scans, to micro-scale pathology slides, to nano-scale proteins and genes, there are challenges associated with analyzing, combining, and correlating these data to make diagnostic, prognostic, and theranostic predictions [2–4]. Computerized image analysis and data integration methods have the potential to improve our understanding of the relationship between these heterogeneous multi-format, multi-scale data to better predict disease outcomes and treatment responses.

## Computer-based Image Analysis

Advances in imaging hardware and computational processing have catalyzed the growth of digital imaging and computer-based image analysis in pathology. Digitization of entire glass slides (whole-slide imaging) has increased the amount of morphologic data that can be obtained from tissue [3]. Whole-slide imaging has also aided pathologists with automated field selection and has begun to allow pathologists to supplement steps in image analysis (i.e., feature extraction, feature selection, dimensionality reduction, and classification) with automated machine-learning algorithms to minimize subjectivity and augment quality assurance [3,5,6].

One such tool, developed, evaluated, and applied by Beck et al., is an unbiased image analysis system called C-Path [7]. C-Path has been used to identify feature sets in tissue microarrays to predict 5-year survival of patients with breast carcinoma. Using a machine-learning algorithm and thousands of morphologic descriptors, the C-Path prognostic model accurately predicted good and poor prognosis patients and identified clinically significant morphologic features, some of which were not previously recognizable using traditional quantitative pathology techniques. Although the molecular basis for the prognositically significant morphologic phenotypes has yet to be elucidated, and the effectiveness of computer-aided pathological interpretation has yet to be established on whole-slide images and tested on a diverse set of images, this approach shows great potential because it has predicted survival outcomes with a high degree of statistical significance and has the potential for further refinement. This example illustrates the potential for using automated, unbiased image analysis and machine-learning systems for producing standardized, objective, reproducible results that could eventually support clinical practice [8].

## Heterogeneous Data Integration

Advances in computational processing have enabled quantitative integration of heterogeneous, multi-format, multi-scale data-particularly imaging and genomic data [2,9–12].

In one of the first applications to combine imaging and non-imaging (protein expression) data, Lee and Madabhushi developed a Generalized Fusion Framework (GFF) to integrate the micro-scale morphological features obtained from digital histopathology slides with nano-scale protein expression measurements from mass spectrometry [13]. This GFF was created to observe whether quantitative integration of image-based signatures from digital histopathology slides with corresponding peptide measurements from mass spectrometry could be used to differentiate prostate cancer progressors with prostate cancer non-progressors. The challenge of integrating this multi-scale, multi-modal, multi-protocol data was overcome by combining the 3 data modalities (architectural histopathology features, morphological histopathology features, and m/z mass spectrometry features in 51, 100, and 570 dimensions, respectively) into a common low-dimensional meta-space projection with 3 dimensions using principal component analysis. This projection was then normalized, concatenated, and reduced a second time with principal component analysis to yield the low-dimensional integration product of the original high-dimensional data. Results reflected the suitability of using this GFF to integrate heterogeneous multi-format, multi-scale data for differentiating between patients with different disease profiles.

Later applications by Madabhushi et al., have explored additional methods for combining data modalities beyond principal component analysis (e.g., non-linear dimensionality reduction methods) and correlations between disease and markers in digital pathology [10], gene and protein expression [11], spectroscopy [12,14], ultrasound [15], and MRI [9,14,16].

## Future Directions

While computer-based image analysis, heterogeneous data integration methods, and computer-aided prognostics are currently demonstrating their efficacy in the pre-operative or pre-therapeutic cancer population, they will inevitably have applicability in other fields.

In cardiovascular medicine, for instance, large amounts of macro-scale heart morphology and phenotype data (from MRI, hemodynamics, and echocardiograms), micro-scale whole-slide imaging data (from biopsies, donors, explants, and device placements), and nano-scale gene expression and transcriptome data are being collected at several institutions for clinical and research purposes [17]. Because typical cardiac pathology scoring systems are rather rudimentary, such as the Dallas criteria for myocarditis [18] and the International Society for Heart and Lung Transplantation scoring of rejection in cardiac allografts [19], there is rich opportunity for computer-aided interpretation and multi-modality integration to provide new insights into myocardial disease mechanisms, severity and prognosis. As with the oncology applications described above, a key step in these myocardial applications will be correlation with clinical outcomes and current clinical reference standards. As heterogeneous data integration tools become increasingly sophisticated and validated, they could provide a rational basis for the identification of interpatient distinctions necessary for greater individualization of therapeutics.

Computers are becoming increasingly ready to supplement and enhance imaging (MRI, ultrasound), morphologic information (tissue), and molecular classification (whole-genome sequencing, expression profiling, proteomics, and metabolomics) with diagnostic, prognostic, and theragnostic predictions [8]. These computer-based tools for heterogeneous data integration have begun to demonstrate their effectiveness in large retrospective studies and will soon be ready for prospective, multi-institutional validation studies as the next step before adoption into clinical practice.
